# TRIM59 loss in M2 macrophages promotes melanoma migration and invasion by upregulating MMP-9 and Madcam1

**DOI:** 10.18632/aging.102351

**Published:** 2019-10-10

**Authors:** Yuan Tian, Yantong Guo, Pei Zhu, Dongxu Zhang, Shanshan Liu, Mengyan Tang, Yuanxin Wang, Zheng Jin, Dong Li, Dongmei Yan, Guiying Li, Xun Zhu

**Affiliations:** 1 Key Laboratory for Molecular Enzymology and Engineering of the Ministry of Education, College of Life Sciences, Jilin University, Changchun, China; 2 Key Laboratory of Pathobiology, Ministry of Education, College of Basic Medical Sciences, Jilin University, Changchun, China; 3 Department of Immunology, Jilin University, Changchun, China

**Keywords:** TRIM59, tumor-associated-macrophages, M2 phenotype, melanoma, metastasis

## Abstract

The culture supernatant from macrophages overexpressing TRIM59 has a cytotoxic effect on melanoma, but the mechanism remains unclear. To investigate whether deletion of TRIM59 in macrophages affects the metastatic potential of melanoma cells, we polarized control and TRIM59-deficient bone marrow-derived macrophages to the M2 phenotype and collected the respective conditioned media (CM). Exposure to CM from TRIM59^-/-^-M2 cultures significantly promoted migration and invasion by B16-F0 and B16-F10 cells. Cytokine profiling indicated a ~15-fold increase in TNF-α production in CM from TRIM59^-/-^-M2 cultures, and neutralizing TNF-α activity abrogated the referred stimulatory effects on cell motility. Transcriptome analysis revealed significant upregulation of *MMP-9* and *Madcam1* in melanoma cells exposed to TRIM59^-/-^-M2 CM. Inhibitory experiments determined that these changes were also TNF-α-dependent and mediated by activation of ERK signaling. Independent knockdown of *MMP9* and *Madcam1* in B16-F10 cells impeded epithelial-mesenchymal transition and inhibited subcutaneous tumor growth and formation of metastatic lung nodules in vivo. These data suggest TRIM59 expression attenuates the tumor-promoting effect of tumor-associated macrophages, most of which resemble the M2 phenotype. Moreover, they highlight the relevance of TRIM59 in macrophages as a potential regulator of tumor metastasis and suggest TRIM59 could serve as a novel target for cancer immunotherapy.

## INTRODUCTION

Melanoma is the most aggressive form of skin cancer; it originates from the malignant transformation of melanocytes, and its incidence is growing faster than that of any other type of cancer [1–3]. Although treatments, including surgery, chemotherapy, and radiotherapy, are often successful, many melanoma patients still die from distal metastasis [4]. In the early stages of metastasis, tumor cells acquire migratory and invasive capabilities that allow them to move through the surrounding stroma, reach the circulation, and infiltrate and colonize distant tissues and organs [5]. Because metastatic spread is the main cause of death in melanoma patients [6–9], a better understanding of this process is paramount to improve early diagnosis and develop effective therapies.

The tumor microenvironment is characterized by complex cellular interactions among tumor cells, non-malignant resident cells, immune cells, endothelial cells, fibroblasts, and structural matricellular components [10]. Among the immune cells recruited to the tumor site, tumor associated macrophages (TAMs) are key determinants of intra-tumor immune status and modulate as well cancer cell-stromal interactions [11]. Macrophages are generally classified into two subsets: classical M1macrophages with antitumor properties, and alternative M2 macrophages with tumor-supporting functions. Most TAMs have a pronounced M2 phenotype, which promotes tumor growth, metastasis, and angiogenesis [11–13]. Thus, elucidating the mechanisms by which TAMs sustain tumor growth and facilitate metastasis should help improve or design immuno-based strategies against cancer.

TRIM59, also known as mouse ring finger protein 1 (Mrf1), belongs to the evolutionarily conserved tripartite motif (TRIM) family of proteins [14]. Most TRIM members possess ubiquitin ligase activity and are involved in many critical processes, such as immunity, antiviral proliferation, transcriptional regulation, cell differentiation, and cancer [15, 16]. At present, the role of TRIM59 is still controversial. Several studies reported that TRIM59 is upregulated in various tumors, such as breast, gastric, colon, lung, and cervical cancer, contributing to tumor proliferation, metastasis, and angiogenesis [16–18]. A study indicated that TRIM59 may suppress RIG-I-like receptor-induced activation of interferon-regulatory factors (IRFs) and NF-κB via interaction with evolutionarily conserved signaling intermediate in Toll pathways (ECSIT), suggesting that TRIM59 may serve as a multifunctional regulator of innate immunity [19].

Our previous work showed that TRIM59 may be an essential molecule underlying the cytotoxic effect of bacillus Calmette Guérin (BCG) -activated macrophages on MCA207 fibrosarcoma cells [20]. In preliminary experiments, we also observed that the supernatant of RAW264.7 cells overexpressing TRIM59 had a strong cytotoxic effect on mouse B16 melanoma cell lines, and this effect was attenuated by a TRIM59-blockingantibody (data not shown). However, the mechanism(s) by which TRIM59 expression in macrophages, and especially in TAMs, influences tumor cell activity and survival remains unclear.

In this study, we generated conditional KO mice with myeloid-specific deletion of TRIM59 (TRIM59-CKO mice) and evaluated the effects of TRIM59^-^/^-^-M2 macrophages on the metastatic behavior of melanoma B16-F0 and B16-F10 cells. B16-F0 is the parent cell line. B16-F10 was obtained by a ten-time selective procedure. B16-F10 cell has stronger migration and invasion ability than B16-F0 cell, and B16-F10 cell is also more capable of forming lung metastatic nodules than B16-F0 cell. To this end, mouse B16 melanoma cell lines were exposed to conditioned media (CM) from bone marrow-derived, TRIM59^-^/^-^ mouse macrophages differentiated into the M2 phenotype, and migration/invasion assays were conducted in vitro. In addition, subcutaneous tumor growth and formation of lung metastases of melanoma cells were evaluated in TRIM59-CKO animals. Our results support and expand previous research suggesting that TRIM59 expression in macrophages critically modulates metastasis in tumor cells, and may be a valuable target for cancer immunotherapy.

## RESULTS

### Melanoma growth is enhanced in mice bearing TRIM59^-/-^ macrophages

**Figure 1:**
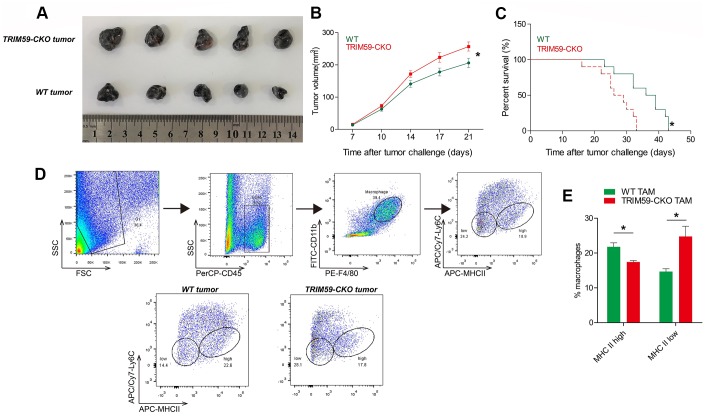
**TRIM59^-/-^ macrophages promote B16-F10 tumor growth.** (**A**) Representative images of B16-F10 tumors from three independent experiments. Wild-type mice or TRIM59-CKO mice were inoculated subcutaneously with B16-F10 cells, and sacrificed 28 days later. *n* = 5 mice per group. (**B**) Mean tumor volume in the different experimental groups. (**C**) Overall survival in WT mice and TRIM59-CKO mice implanted with B16 melanoma allografts. *n* = 10 mice per group. (**D**) Phenotypic screening of TAMs. Representative FACS plots from WT and TRIM59-CKO mouse macrophages. (**E**) Flow cytometry analysis of macrophage subpopulations based on Ly6C and MHCII expression. Data are represented as mean ± SD. *p<0.05, compared to WT.

Preliminary experiments showed that CM from macrophages overexpressing TRIM59 had cytotoxic effects on B16-F0 cells (data not shown). To determine whether TRIM59 expression in macrophages influences melanoma growth and migration/invasion, we subcutaneously injected B16-F10 mouse melanoma cells into WT and myeloid-specific TRIM59-CKO mice. Mean tumor volume (Figure 1A–1B) was significantly greater in TRIM59-CKO mice compared to WT mice. In addition, overall survival was significantly decreased in TRIM59-CKO mice (Figure 1C). Following tumor resection, TAMs phenotypes were studied by flow cytometry. A previous study classified TAMs into different subpopulations based on the expression of Ly6C and MHCII. Accordingly, Ly6C^lo^MHCII^hi^ TAMs are M1-like macrophages, and Ly6C^lo^MHCII^lo^ TAMs are M2-like macrophages [21, 22]. Using this categorization, we found that TAMs inTRIM59-CKO mice presented mainly the M2 phenotype (Figure 1D–1E).

### TRIM59^-/-^-M2 macrophages promote melanoma cell proliferation, migration, and invasion in vitro

**Figure 2:**
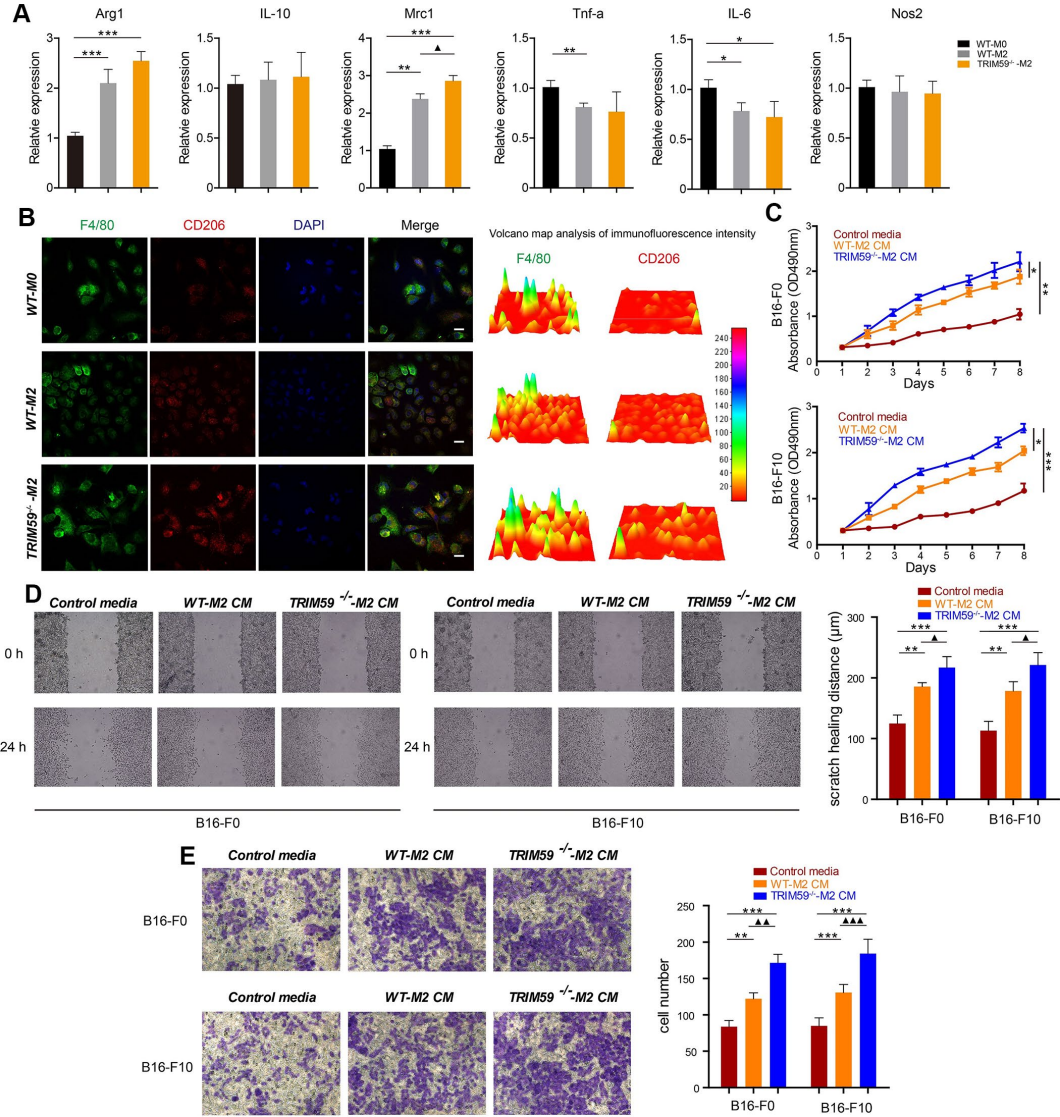
**TRIM59^-/-^-M2 macrophage CM promotes melanoma cell migration and invasion.** (**A**) The expression of Arg1, IL-10, Mrc1, TNF-α, IL-6, and NOS2 was detected by qRT-PCR. Data are represented as mean ± SD. *p<0.05, **p<0.01, ***p<0.001, compared with the WT-M0 group; ▲*p*<0.05, TRIM59^-/-^-M2 vs. WT-M2. (**B**) Immunofluorescent detection of F4/80 and CD206. Fluorescence intensity was analyzed by a 3D surface plot using ImageJ. Scale bars = 100 μm. (**C**) Proliferation assay results from B16-F0 and B16-F10 cells treated with control media, WT-M2 CM, or TRIM59^-/-^-M2 CM. Data are represented as mean ± SD. *p<0.05, **p<0.01, ***p<0.001, compared withTRIM59^-/-^-M2 CM. (**D**) Representative images from wound healing (cell migration) assays and data quantification. Data are represented as mean ± SD. **p<0.01, ***p<0.001, compared with control media; ▲*p*<0.05, TRIM59^-/-^-M2 CM vs. WT-M2 CM. (**E**) Representative images from transwell (cell invasion) assays and data quantification. Data are represented as mean ± SD. **p<0.01, ***p<0.001, compared with control media; ▲▲*p*<0.01, ▲▲▲*p*<0.001, TRIM59^-/-^-M2 CM vs. WT-M2 CM.

In light of the above in vivo results, we carried out in vitro experiments to investigate the effects of macrophage TRIM59 deficiency on melanoma cell growth, migration, and invasion. To this end, we induced the differentiation of bone marrow-derived macrophages (BMDMs) from WT and TRIM59-CKO mice into M2 macrophages. The M2 phenotype was verified through RT-PCR by high expression of the M2 markers *Arg-1*, *IL-10*, and *Mrc1* and low expression of the M1 markers *TNF-α*, *IL-6*, and *NOS2* (Figure 2A). FACS analysis further showed that TRIM59^-/-^-M2 macrophages exhibited higher CD206 expression and lower MHCII expression than WT-M0 macrophages and WT-M2 macrophages. The expression of CD206 in WT-M2 macrophages was also higher than in non-polarized (M0) WT macrophages (Supplementary Figure 2A). We also performed immunofluorescence to detect F4/80 (macrophage marker) and CD206 (M2 phenotype macrophage marker), the results confirmed that M2 macrophages expressed high levels of CD206 (Figure 2B).

Next, melanoma B16-F0 and B16-F10 cells were incubated with CM from WT-M2 or TRIM59^-/-^-M2 macrophages. As shown in Figure 2C, although cell proliferation was promoted by either CM, the effect was significantly larger after incubation with CM from TRIM59^-/-^-M2 macrophages. Moreover, the latter also enhanced the migratory capability of B16-F0 and B16-F10 cells, measured through wound-healing assays, whereas WT-M2-CM had little effect (Figure 2D). Similarly, incubation with TRIM59^-/-^-M2 macrophage CM significantly increased invasion of B16-F0 and B16-F10cells through Matrigel-coated membranes in transwell assays, while WT-M2 macrophage CM had only a slight effect (Figure 2E).

Additional assays were conducted in human melanoma A375 cells pre-treated with CM from M2-polarized TRIM59^+/+^ and TRIM59^-/-^ human THP1 macrophages. Results showed that CM from TRIM59^-/-^-THP1 cells enhanced migration and invasion of A375cells, whereas control THP1 CM had little effect (Supplementary Figure 1B–1C). In addition, we detected the cytotoxic effects of the CM from THP1 which overexpressed TRIM59 on A375 cells. The results are THP1 conditioned medium has notably little cytotoxicity on A375 while CM from TRIM59 ovexpressed THP1 cells showed prominent cytotoxicity in killing A375 (Supplementary Figure 1D). Overall, these results suggest that loss of TRIM59 is correlated with a protumoral function of M2 macrophages.

### High TNF-α production by TRIM59^-/-^-M2 macrophages promotes MMP9 and Madcam1 expression in melanoma cells

**Figure 3:**
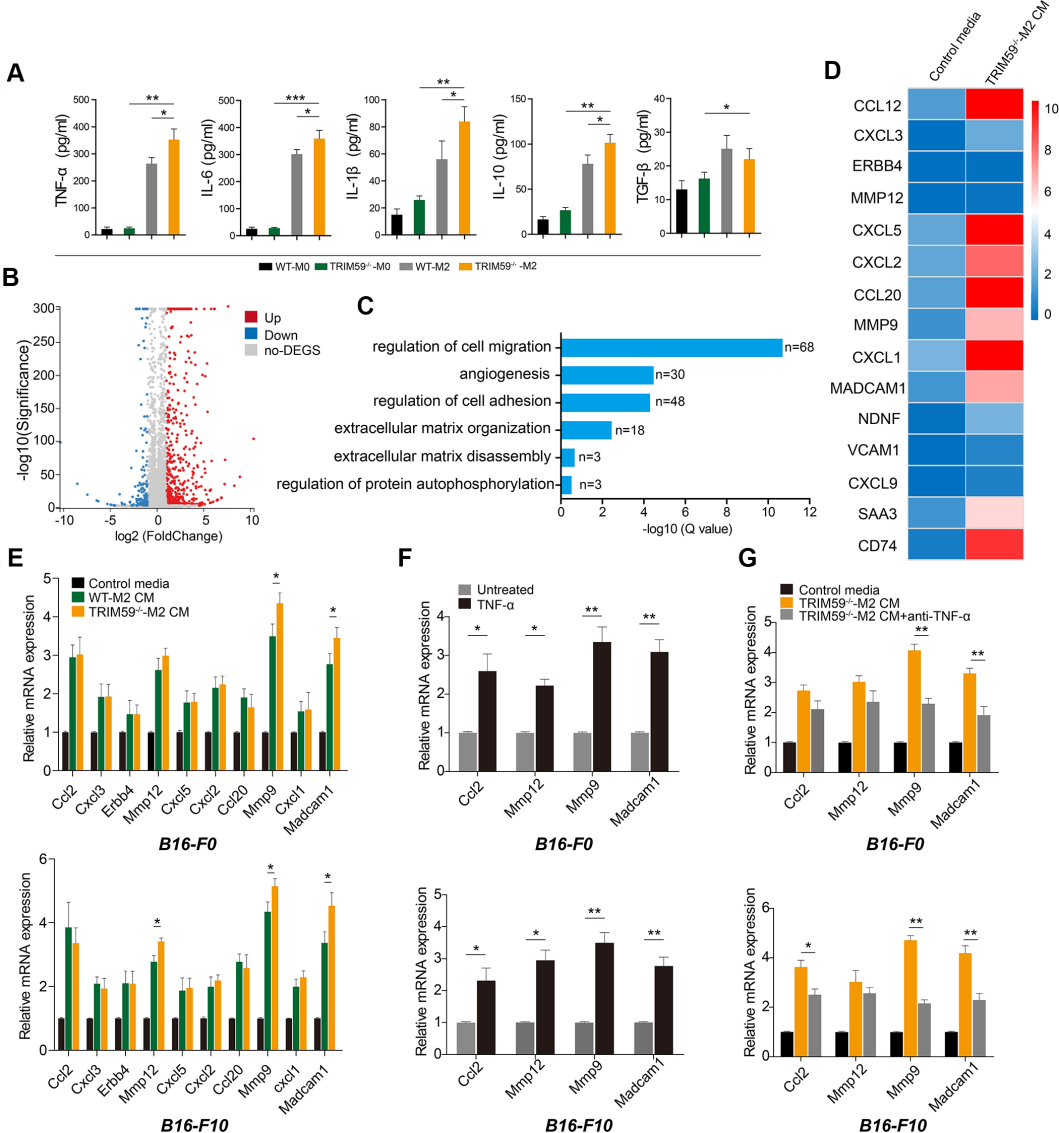
**CM from TRIM59^-/-^-M2 contains TNF-α and induces expression of *ccl2*, *MMP-12*, *MMP-9*, and *Madcam1* in melanoma cells.** (**A**) ELISA detection of cytokines in culture supernatants from WT-M0, TRIM59^-/-^-M0, WT-M2, and TRIM59^-/-^-M2 macrophages. Data are represented as mean ± SD. *p<0.05, **p<0.01, ***p<0.001, compared with the TRIM59^-/-^-M2 group. (**B**) Scatter plots showing DEGs detected in B16-F10 cells treated with TRIM59^-/-^-M2 CM. (**C**) GO enrichment analysis of DEGs identified in B16-F10 cells exposed to TRIM59^-/-^-M2 CM. (**D**) Top 15 DEGs. (**E**) qRT-PCR detection of the top 10 DEGs in cells treated with control media, WT-M2 CM, orTRIM59^-/-^-M2 CM. Data are represented as mean ± SD. *p<0.05, compared with the WT-M2 CM group. (**F**) qRT-PCR detection of *ccl2*, *MMP-12*, *MMP-9*, and *Madcam1* expression in B16-F0 and B16-F10 cells treated with or without TNF-α. Data are represented as mean ± SD. *p<0.05, **p<0.01, compared with untreated cells. (**G**) qRT-PCR detection of *ccl2*, *MMP-12*, *MMP-9*, and *Madcam1* expression in B16-F0 and B16-F10 cells treated with control media, TRIM59^-/-^-M2 CM, or TRIM59^-/-^-M2 CM containing a neutralizing TNF-α antibody. Data are represented as mean ± SD. *p<0.05, **p<0.01, compared with untreated cells.

To investigate the mechanism by which TRIM59^-/-^-M2 macrophage CM promoted the migration and invasion of melanoma cells, we used ELISA to detect the concentrations of relevant cytokines in M2 macrophage supernatants. We found a ~15-fold increase in tumor necrosis factor-α (TNF-α) contents in CM from M2 macrophages, relative to CM from non-differentiated (M0) cells. In turn, TNF-α levels in CM from TRIM59^-/-^-M2 macrophages were significantly higher than in CM from WT-M2 macrophages. At the same time, we observed that synthesis of pro-inflammatory factors such as IL-6 and IL-1β was increased in M2 macrophages, but there was no significant difference between TRIM59^-/-^-M2 CM and WT-M2 CM. IL- 10 was also increased in M2 macrophages, while TGF-β was basically unchanged (Figure 3A).

Next, we compared gene expression patterns between B16-F10 cells cultured with either TRIM59^-/-^-M2 macrophage CM. A total of 813 differentially expressed genes (DEGs), comprising 648 upregulated and 165 downregulated transcripts, were detected by transcriptome analysis (Figure 3B). These DEGs were classified according to gene ontology (GO) analysis (Figure 3C). 68 DEGs were associated with regulation of cell migration; these included genes encoding chemokines (*ccl2*, *cxcl3*, *cxcl5*, *ccl20*, and *cxcl1*), matrix metalloproteinases *(MMPs), i.e. MMP-9* and*MMP-12*, and the adhesion molecule *Madcam1* (Figure 3D). The transcriptome sequencing results were validated by quantitative RT-PCR. Compared with the control group, *ccl2,* *MMP-12*, *MMP-9*, and *Madcam1* gene levels were significantly up-regulated in B16-F0 and B16-F10 cells cultured with CM from TRIM59^-/-^-M2 macrophages (Figure 3E).

To reconcile the above ELISA and gene expression data, we tested the effects of TNF-α on the expression of some key DEGs. Addition of recombinant mouse TNF-α (100 ng/ml) upregulated *ccl2*, *MMP12*, *MMP9*, and *Madcam1* mRNA in both B16-F0 and B16-F10 cells (Figure 3F). Conversely, blockade of TNF-α synthesis in TRIM59^-/-^-M2 macrophage cultures using a neutralizing antibody significantly negated *MMP-9* and *Madcam1* induction in both B16-F0 and B16-F10 cells exposed to the corresponding CM (Figure 3G). Taken together, these results suggest that TNF-α production by TRIM59^-/-^-M2 macrophages promotes the expression of *MMP-9* and *Madcam1* in B16-F0 and B16-F10 tumor cells, enhancing their metastatic potential.

### Conditioned media from TRIM59^-/-^-M2 macrophages activates the ERK pathway in melanoma cells

**Figure 4:**
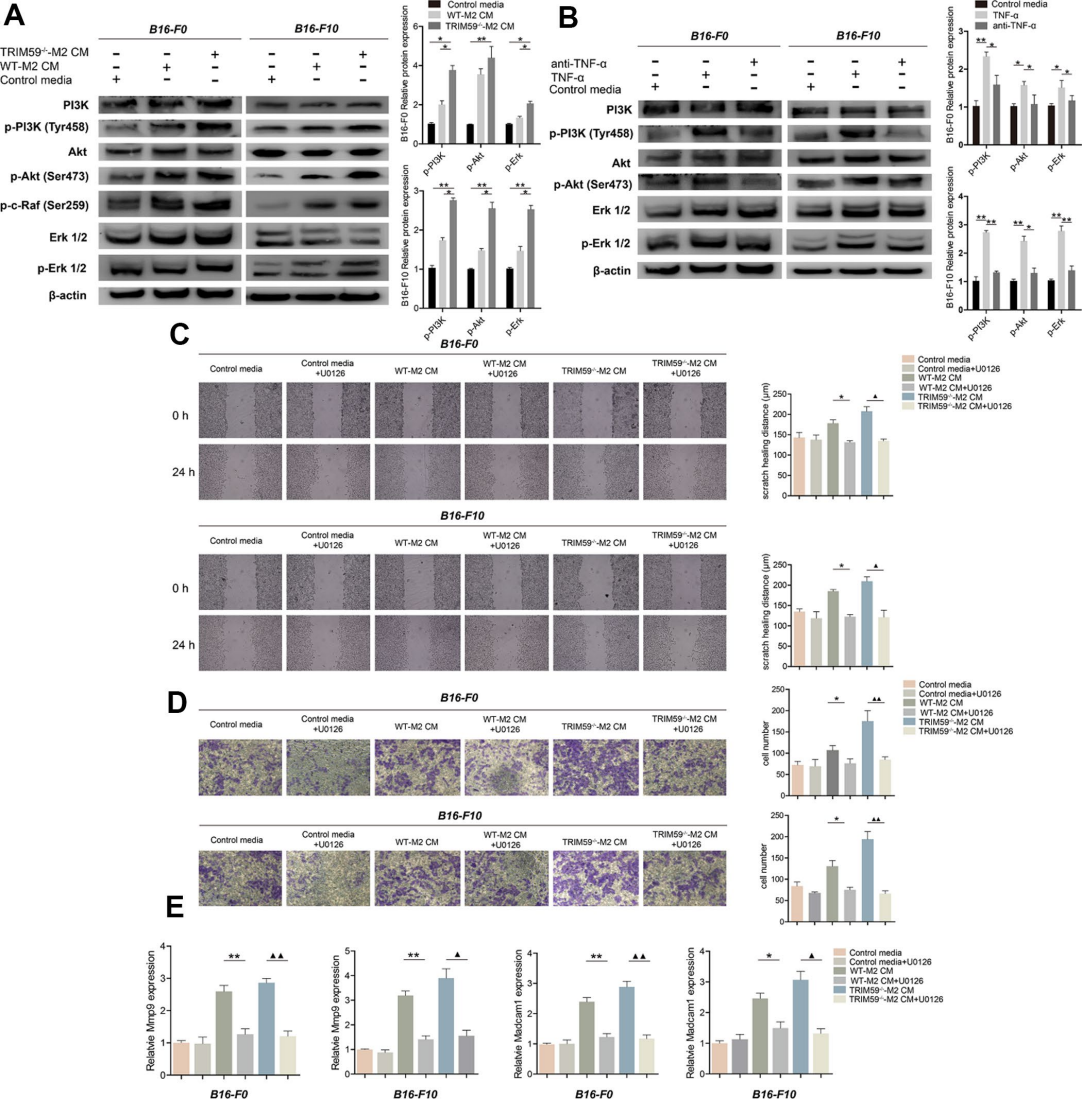
**TRIM59^-/-^-M2 macrophage CM activates the PI3K-Akt and ERK pathways in melanoma cells.** (**A**) Western blot detection of signaling proteins related to the PI3K and ERK pathways in B16-F0 and B16-F10 cells treated with control media, WT-M2 CM, or TRIM59^-/-^-M2 CM. Data are represented as mean ± SD. *p<0.05, **p<0.01, compared with the TRIM59^-/-^-M2 CM group. (**B**) Western blot detection of signaling proteins related to the PI3K and ERK pathways in B16-F0 and B16-F10 cells treated with control media, TNF-α, or a TNF-α antibody. Data are represented as mean ± SD. *p<0.05, **p<0.01, compared with the TNF-α group. (**C**) Transwell assays results showing the invasive ability of B16-F0 and B16-F10 cells in response to CM from M2 macrophage cultures in the presence of the ERK inhibitor U0126. (**D**) Wound healing assay results showing the migratory ability of B16-F0 and B16-F10 cells in response to CM from M2 macrophage cultures in the presence of the ERK inhibitor U0126. (**E**) qRT-PCR evaluation of *MMP-9* and *Madcam1* expression in B16-F0 and B16-F10 cells exposed to CM from M2 macrophage cultures in the presence of the ERK inhibitor U0126. Data are represented as mean ± SD. *p<0.05, **p<0.01, WT-M2 CM vs. WT-M2 CM plus U0126; **▲***p*<0.05, **▲▲***p*<0.01, TRIM59^-/-^-M2 CM vs. TRIM59^-/-^-M2 CM plus U0126.

To further assess the molecular changes underpinning enhanced migration and invasion of B16-F0 and B16-F10 cells exposed to CM from M2-polarized macrophages, the activation status of the PI3K and the ERK signaling pathways was examined by western blotting. Compared with control media, CM from M2 macrophages induced pronounced increases in PI3K phosphorylation (Tyr458), Akt phosphorylation (Ser473), c-raf phosphorylation (Ser289), and ERK1/2 phosphorylation (Thr202/Tyr204) in both B16-F0 and B16-F10 cells. These changes were especially marked in melanoma cells exposed to CM from TRIM59^-/-^-M2macrophages, compared with CM from WT-M2 cells (Figure 4A). ERK signaling is an important regulator of cancer cell proliferation, migration, and invasion in many tumor types. We verified that ERK signaling is activated by TNF-α (100 ng/ml) in B16-F0 and B16-F10 cells, while antibody-mediated TNF-α inhibition significantly decreased this activation (Figure 4B). Furthermore, in both cell types inhibition of ERK1/2 with U0126 (10 μM) suppressed migration and invasion induced by CM from M2 macrophages (either WT orTRIM59^-/-^; Figure 4C–4D), while the Akt inhibitor MK2206 (10 μM), tested in B16-F10 cells, was less effective (Supplementary Figure 3A–3C). In turn, ERK signaling inhibition in B16-F0 and B16-F10 cells abolished the upregulation of *MMP-9* and *Madcam1* mRNA observed after stimulation with CM from M2 macrophages (Figure 4E). These data suggest that TRIM59 deficiency triggers TNF-α production by M2 macrophages, which promotes migration and invasion of melanoma cells mainly via activation of the ERK pathway and subsequent upregulation of *MMP-9* and *Madcam1*.

### MMP-9 and Madcam1 knockdown decreases migration and invasion and suppresses epithelial-mesenchymal transition in melanoma cells

**Figure 5:**
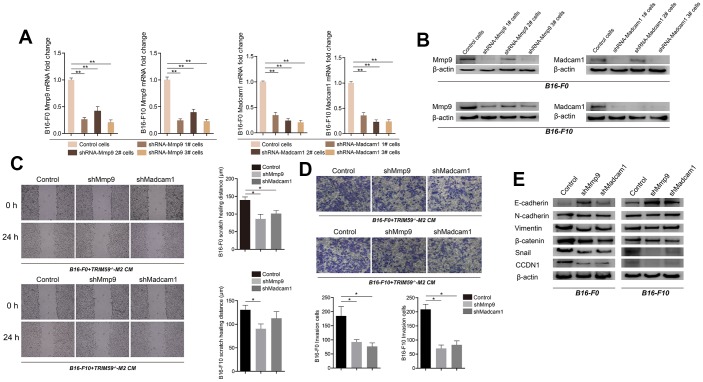
**Knockdown of *MMP-9* or *Madcam1* inhibits migration, invasion, and EMT in melanoma cells.** (**A**) Expression of *MMP-9* and *Madcam1* in B16-F0 and B16-F10 cells transfected with *MMP-9*-shRNA, *Madcam1*-shRNA or scrambled (control)-shRNA, as assessed by qRT-PCR. Data are represented as mean ± SD. **p<0.01, compared with control cells. (**B**) Expression of MMP9 and Madcam1 in B16-F0 and B16-F10 cells transfected with *MMP-9*-shRNA, *Madcam1*-shRNA, or scrambled (control)-shRNA, as assessed by western blotting. A representative image from three independent experiments is shown. (**C**) Representative images of cell migration assays conducted on B16-F10 cells transfected with control shRNA, *MMP-9*-shRNA, or *Madcam1*-shRNA. Data are represented as mean ± SD. *p<0.05, compared with control cells. (**D**) Representative images of transwell invasion assays conducted on B16-F10 cells transfected with control shRNA, *MMP-9*-shRNA, or *Madcam1*-shRNA. Data are represented as mean ± SD. *p<0.05, compared with control cells. (**E**) Western blot detection of EMT-associated proteins, including E-cadherin, N-cadherin, and vimentin, in B16-F0 and B16-F10 cells after shRNA-mediated downregulation of *MMP-9* or *Madcam1*.

To verify the role of *MMP-9* and *Madcam1* in B16-F0 and B16-F10 cells, specific shRNAs were used to inhibit their expression. Transfection with *MMP-9*-shRNA decreased basal *MMP-9* mRNA levels by ~3- and 3.5-fold in B16-F0 and B16-F10 cells, respectively. In turn, *Madcam1*-shRNA transfection reduced basal *Madcam1* mRNA levels by ~ 4-fold in both B16-F0 and B16-F10 cells (Figure 5A). Decreased MMP-9 and Madcam1 protein levels were confirmed by western blotting (Figure 5B). Knockdown of either *MMP-9* or *Madcam1* significantly decreased the migration of B16-F0 and B16-F10 cells in response to CM from TRIM59^-/-^-M2 macrophages (Figure 5C). Similar effects were observed on transwell invasion assays (Figure 5D).

Based on these findings, we used western blotting to test the hypothesis that *MMP-9* and *Madcam1* might regulate epithelial-mesenchymal transition (EMT) in B16 cells. Consistent with EMT repression, results showed that *MMP-9* or *Madcam1* knockdown up-regulated E-cadherin and downregulated N-cadherin, vimentin, β-catenin, snail, and CCDN1 in B16-F0 and B16-F10 cells.

### MMP-9 and Madcam1 knockdown inhibits B16-F10 tumor progression and metastatic burden

**Figure 6:**
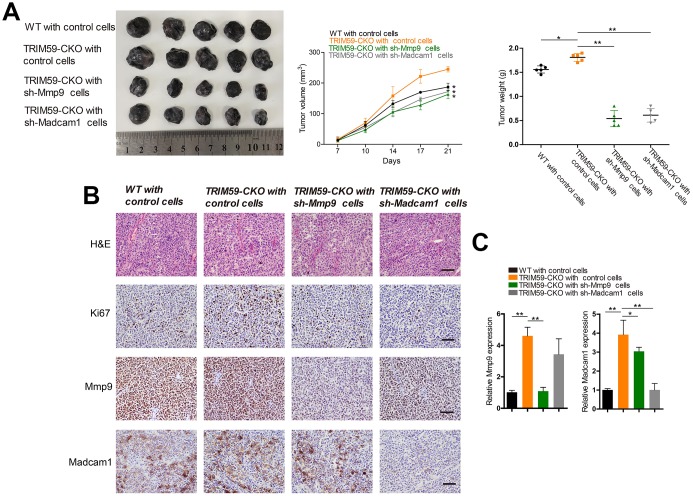
**Knockdown of *MMP-9* or *Madcam1* inhibits melanoma growth *in vivo*.** (**A**) Representative image of B16-F10 tumors from three independent experiments. WT or TRIM59-CKO mice were inoculated subcutaneously with B16-F10 cells transfected with control shRNA, *MMP-9*-shRNA, or *Madcam1*-shRNA. Tumor volumes were measured twice a week, and tumor weights estimated on sacrifice at day 28. *n* = 5 mice per group. Data are represented as mean ± SD. *p<0.05, **p<0.01, compared to tumors generated by shRNA control B16 cells in TRIM59-CKO mice. (**B**) Representative H&E staining images and IHC detection of Ki67, MMP-9, and Madcam1 in B16-F10 allografts (original magnification × 200). (**C**) Detection of *MMP-9* and *Madcam1* mRNA expression in B16-F10 allografts by qRT-PCR. Data are represented as mean ± SD. *p<0.05, **p<0.01, compared to tumors generated by shRNA control B16 cells in TRIM59-CKO mice.

To assess the effects of *MMP-9* and *Madcam1* deletion on tumor growth *in vivo*, we subcutaneously injected B16-F10 cells transfected with shRNA-*MMP-9*, shRNA-*Madcam1*, or scrambled control-shRNA into WT or TRIM59-CKO mice. B16-F10 control cells in TRIM59-CKO mice produced the largest tumors in WT with scrambled control-shRNA group, TRIM59-CKO with scrambled control-shRNA group, TRIM59-CKO with shRNA-*MMP-9* group and TRIM59-CKO with shRNA-*Madcam1* group, while those generated in the shRNA-*MMP-9* and shRNA-*Madcam1* groups were significantly smaller (Figure 6A). This was consistent with decreased proliferation, indicated by reduced Ki67 immunostaining, in tumors generated by sh-*MMP-9*- and sh-*Madcam1*-transfected B16-F10cells (Figure 6B). Both immunohistochemistry and qRT-PCR confirmed decreased MMP-9 and Madcam1 expression in tumor samples derived from cells in which *MMP9* or *Madcam1* were knocked down (Figure 6B–6C).

**Figure 7:**
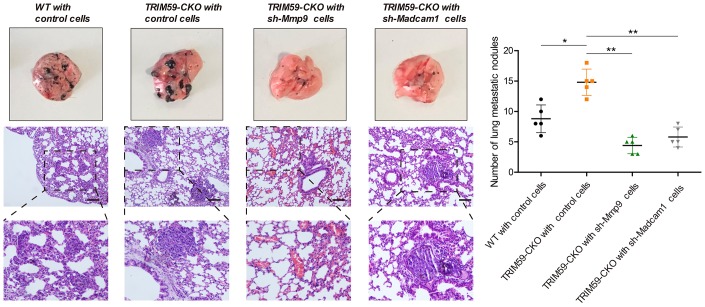
**MMP-9 or Madcam1 silencing decreases pulmonary metastasis of melanoma cells.** Representative macroscopic and H&E images of mouse lungs colonized by metastatic B16-F10 melanoma cells. Scale bar = 20 μm. Quantification of metastatic foci (mean ± SD, *n* = 5) for each experimental condition is shown. Data are represented as mean ± SD. *p<0.05, **p<0.01, compared with shRNA control B16-F10 cells inoculated in TRIM59-CKO mice.

To evaluate potential contributions of MMP-9 and Madcam1 to metastasis formation by melanoma cells, B16-F10 cells transfected with shRNA-*MMP-9*, shRNA-*Madcam1*, or scrambled control-shRNA were injected intravenously into WT or TRIM59-CKO mice. Although lung metastatic nodules developed in all mice, they were more numerous in TRIM59-CKO mice injected with control B16-F10 cells. In contrast, lung metastatic nodules were less numerous in the shRNA-*MMP-9* and shRNA-*Madcam1* groups (Figure 7). These results indicated that *MMP-9* or *Madcam1* deletion attenuates lung metastatic colonization by melanoma cells facilitated by myeloid-specific TRIM59 knockdown.

## DISCUSSION

This study shows that the growth of B16 melanoma allografts was stimulated by myeloid-specific TRIM59 knockdown in host mice, indicating that TRIM59 expression in TAMs may prevent or attenuate melanoma progression. Phenotypic analysis showed that the M2 phenotype prevailed among isolated TRIM59^-^/^-^ TAMs. Exposure to TRIM59^-/-^-M2 macrophage supernatant promoted migration and invasion of B16 melanoma cells, and inhibitory experiments indicated that overexpression of TNF-α by TRIM59-deficient M2 macrophages and activation of ERK, and perhaps PI3K, signaling in B16 melanoma cells mediated these effects. RNA sequencing identified a large number of DEGs in B16-F10 cells exposed to TRIM59^-/-^-M2 macrophage CM. Among the top 15 DEGs, we selected *MMP-9* and *Madcam1*, i.e. two genes involved in cell motility processes, and confirmed their regulation by TNF-α-induced ERK activation. Independent knockdown of either *MMP-9* or *Madcam1* by shRNA in melanoma cells inhibited EMT and invasion induced by TRIM59^-/-^-M2 macrophage CM, and attenuated the growth of subcutaneous B16-F10 tumors and reduced lung metastasis burden in TRIM59-CKO-mice.

Originally described as a circulating factor that can cause necrosis in tumors, TNF-α is now known to be a key regulator of inflammatory and immune responses [23]. TNF-α exerts multiple, context-dependent actions, and has been deemed a tumor-promoting factor for its ability to promote tumor survival by indirect mechanisms (e.g. immunosuppression) or direct effects on cancer cells (e.g. induction of EMT) [24]. Inflammation within the tumor microenvironment critically contributes to tumor development [25]. Accordingly, studies showed that upregulation of TNF-α in endothelial and immune cells promotes early tumor inflammation and stromal interactions that facilitate tumor invasion and metastasis [26–31]. In some tumor types, TNF-α was shown to promote tumor cell migration and metastasis by inducing RAS or c-MYC activation and expression of MMPs (e.g. MMP-3 and 9) [32–36]. TNF-α can also stimulate the transcription of genes encoding endothelial cell adhesion molecules, including E selectin, ICAM-1, VCAM-1, and Madcam1, which are closely related to tumor metastasis and angiogenesis [37–39]. Our study showed that TNF-α promoted the expression of *MMP-9* and *Madcam1* in melanoma cells, which contributed to melanoma growth and metastasis. Because knockdown of *MMP-9* did not alter Madcam1 protein levels, and *Madcam1* silencing did not affect MMP-9 protein expression, we conclude that the effects were independent, and that both proteins are needed for metastatic dissemination of melanoma cells.

TRIM59, a member of the TRIM ubiquitin ligase family, has long been considered a tumor marker in association with tumor progression and metastasis in several tumor types, such as non-small cell lung cancer, gastric cancer, osteosarcoma, and cervical cancer [17, 43–46]. A recent study reported that depletion of TRIM59 suppresses breast cancer metastasis by promoting RNFT1-induced K63 poly ubiquitination and SQSTM1-directed autophagic degradation of PDCD10 [40]. Another study reported that TRIM59 promotes gliomagenesis by inhibiting TC45 dephosphorylation of STAT3 [41]. TRIM59 can also regulate autophagy by modulating both the transcription and ubiquitination of BECN1 [42]. Interestingly, we found that TRIM59 expression is induced in BCG-stimulated macrophages and mediates a cytotoxic effect by direct cell contact on MCA207 fibrosarcoma cells [20]. In the present work, TRIM59 deficiency stimulated the metastatic potential of melanoma cells, suggesting that expression of TRIM59 in macrophages may have antitumor effects. We also found that TRIM59 expression in macrophages did not alter their phenotype, but significantly increased phagocytic activity [47], which may be a mechanism involved in the antitumor response.

M2-like macrophages predominate in the tumor microenvironment, and play a stimulatory role on tumor development and metastasis [48, 49]. In our study, we found that deletion of TRIM59 further enhanced the tumor-promoting actions of M2 macrophages by inducing production and the release of TNF-α. Recent studies indicated the involvement of several ubiquitin ligases, including TRIM family members such as TRIM45, in the regulation of the MAPK signaling pathway (through alterations in AP-1/Elk-1transcriptional activity) and the NF-κB signaling pathways [50–52]. In our study, TRIM59^-^/ ^-^macrophages mediated upregulation of the PI3K and ERK signaling pathways in B16 melanoma cells, promoting *MMP-9* and *Madcam1* expression and stimulating migration and invasion *in vitro*, as well as tumorigenesis, EMT, and metastasis in an *in vivo* tumor model.

In conclusion, our study suggested that expression of TRIM59 in macrophages potentially restricts melanoma progression and metastasis, and its loss has pro-tumoral effects via induction of TNF-α. This evidence highlights that upregulation of TRIM59 in TAMs could be an effective way to attenuate or prevent tumor growth, in melanoma and perhaps other cancers.

## MATERIALS AND METHODS

### Cell culture

B16-F0 and B16-F10 mouse melanoma cells, primary mouse bone marrow-derived macrophages, THP1 cells, and A375 human melanoma cells were maintained in Dulbecco’s modified Eagle’s medium (DMEM) supplemented with 10% fetal bovine serum (FBS) and antibiotics. All cells were cultured in a humidified atmosphere of 5% CO_2_ at 37°C.

### Antibodies and reagents

Anti-PI3K-p85 (cat. no. 4257T), anti-phospho-PI3K-p85 (cat. no. 4228S), anti-Akt (cat. no.4691S), anti-phospho-Akt (cat. no. 4060S), anti-Erk (cat. no. 3552S), anti-phospho-Erk1/2 (cat. no. 4370S), anti-phospho-c-raf (cat. no. 9427S), anti-E-cadherin (cat. no. 3195S), anti-N-cadherin (cat. no. 13116T), anti-vimentin(cat. no. 5741S), anti-β-catenin (cat. no. 8480S), anti-Snail (cat. no. 3879S), anti-cyclinD1 (cat. no. 2978S), anti-β-actin (cat. no. 4970S), anti-MMP-9 (cat. no. 13667S), and anti-TNF-α (cat. no. 11948S) were from Cell Signaling Technology (Danvers, MA, USA). Anti-Madcam1 (cat. no. AF993) was from R&D Systems (Minneapolis, MN, USA). Recombinant mouse IL-4 (cat. no. 214-14) and IL-13 (cat. no. 210-13) were from Peprotech (Rocky Hill, NJ, USA). Recombinant mouse TNF-α (cat. no. abs04259) was from Absin Bioscience Inc. (Shanghai, China). U0126 (cat. no. 1095821), a selective inhibitor of MEK1 and MEK2, and MK2206 (cat. No. 1031320), a selective inhibitor of Akt, were both from Peprotech.

### Animals

All animal studies were conducted in accordance with the guidelines established by the Animal Research Committee of Jilin University. The TRIM59 targeting vector was constructed by flanking the exons of TRIM59 with 2 loxP sequences. TRIM59^Flox/Flox^ (TRIM59^F/F^) mice were produced by homologous recombination using the C57BL/6N ES cell line RENKA (Cyagen Biosciences Inc., Guangzhou, China). TRIM59^F/F^ mice were crossed with Lyz2-Cre transgenic mice (Jackson Laboratories, Bar Harbor, USA) to produce TRIM59^F/F^: Lyz2-Cre (TRIM59-CKO) mice. These mice only knocked out TRIM59 in macrophages. These conventional macrophage TRIM59-KO mice were backcrossed onto the C57BL/6genetic background and used in the present study.

### Tumor models

For subcutaneous tumor implantation, B16-F0 or B16-F10 melanoma cells transfected with control shRNA or shRNAs targeting either *MMP-9* or *Madcam1* were injected subcutaneously (5×10^5^ cells in 150 μl saline) into the sides of 8- to 10-week-old female WT or TRIM59-CKO mice. Tumor volume (in mm^3^) was measured twice a week with a digital caliper and calculated by the following formula: volume = (width)^2^ × length/2. On day 28 post-implantation mice were sacrificed, and tumors were harvested for downstream experiments.

To evaluate metastasis development *in vivo*, B16-F0 or B16-F10 melanoma cells transfected with control shRNA, or shRNAs targeting either *MMP-9* or *Madcam1* were injected intravenously (5 × 10^5^ cells in 100 μl saline) into 8- to 10-week-old female WT or TRIM59-CKO mice. For all animal experiments, at least five mice were randomly selected and included in each experimental group, and all animals used were included in the analysis.

### Bone marrow-derived macrophage (BMDM) induction and conditioned medium preparation

Bone marrow cells were flushed from mouse bones and cultured in DMEM (20% FBS, 2 mM L-glutamine, 50 μM 2-mercaptoethanol) with 40 ng/ml macrophage colony stimulating factor (M-CSF). The medium was changed every two days, and the cells differentiated into BMDMs in one week. To generate M2 macrophages, BMDM cells were cultured with 25 ng/ml IL-4 and 25 ng/ml IL-13 for 48 h, and IL-4 and IL-13 were then removed by thorough washing. M2 cells were further cultured in 2 mL fresh serum-free DMEM for another 24 h to produce the conditioned media from M2 cells. Conditioned media was collected and stored at 80 °C.

### Cell proliferation, wound healing and transwell assays

Cells were cultured in serum-free DMEM for 24 h before seeding at 1 × 10^3^ cells per well in 96-well plates in control media, WT-M2 conditioned media, orTRIM59^-/-^-M2 conditioned media. Cell proliferation was measured using Cell Count Kit-8 (CCK-8) reagent (10 μl was added to each well at the endpoint) at the indicated times according to the manufacturer’s instructions (Dojindo Laboratories, Kumamoto, Japan). In wound healing assays, melanoma cancer cells were seeded into 6-well plates and scraped using a sterile pipette tip. Images were obtained using an inverted microscope at 0 and 24 h and then analyzed by ImageJ software (NIH, Bethesda, MD, USA). For cell invasion assays, melanoma cancer cells were seeded into Matrigel-coated upper chambers of Transwell inserts (Corning, NY, USA). After 24 h of incubation, non-invading cells were scraped off with a cotton swab, and the cells on the bottom of the chamber were fixed, stained, and counted.

### Flow cytometry and immunofluorescence

Flow cytometry and/or immunofluorescence was used to analyze F4/80, CD11b, MHCII, and Ly6C expression in TAM subtypes, and F4/80, MHCII, and CD206 expression in BMDM subtypes. APC/Cy7-Ly6C (557661), PE-F4/80 (565410), and FITC-CD11b (557396) were from BD Biosciences (San Jose, CA, USA); APC-MHCII (107614) and FITC-CD206 (141703) were from BioLegend (San Diego, CA, USA).

### Enzyme-linked immunosorbent assay (ELISA)

Murine TNF-α, IL-6, IL-1b, IL-10, and TGF-β were measured in serum-free CM from WT-M0, WT-M2, TRIM59-CKO-M0, and TRIM59-CKO-M2 cells using murine ELISA kits purchased from eBioscience (San Diego, CA, USA) according to the manufacturer’s instructions. Murine IL-2 was measured in serum free CM from WT-M0, WT-M2, and TRIM59-CKO-M2 cells using murine ELISA kits purchased from R&D Systems (Minneapolis, MN, USA). Absorbance was measured at 450 nm and corrected at 540 nm using a microplate reader. Total protein concentrations in CM were calculated using CurveExpert 1.4. (CurveExpert and GraphExpert Software, Madison, AL). Statistical analysis was performed using Student’s t-test. A p < 0.05 was considered statistically significant.

### Western blot analysis

Cells were harvested and lysed in lysis buffer (50mM Tris-HCl, 1% NP40, 150 mM NaCl, 1mM EDTA, and 1mM PMSF) for 30 min at 4°C. Total cell extracts were separated using 12% sodium dodecyl sulfate-polyacrylamide gels and transferred to polyvinylidene difluoride membranes. The membranes were blocked with 3% bovine serum albumin and incubated with primary antibodies diluted in blocking solution. Signals were visualized using a chemiluminescent substrate (Super Signal West Pico Kit; Thermo Scientific, IL, USA).

### RNA isolation and quantitative real-time PCR

Total RNA was extracted from cultured or sorted macrophages using TRIzol reagent (Takara, Tokyo, Japan) according to the manufacturer’s instructions. cDNA was synthesized and analyzed via quantitative real-time PCR with SYBR Mix. Expression levels of the target genes were normalized to that of the control gene.

### Cell transfection

*MMP-9* and *Madcam1* shRNA plasmids and shRNA negative control were purified and synthesized by Gene-Pharma (Shanghai, China). Transfection was performed using polyethylenimine transfection reagent (Polysciences, Inc., Warrington, PA). RNA sequences were as follows: Sh-*MMP-9*: 5′-3′CCAACUAUGACCA GGAUAATT, 3′-5′UUAUCCUGGUCAUAGUUGGTT; Sh-*Madcam1*: 5′-3′GCUCUUGUUUGCCGAGCUATT, 3′-5′UAGCUCGGCAAACAAGAGCTT.

### Immunohistochemistry

Tumors from WT or TRIM59-CKO mice were fixed for 72 h with 10% buffered formalin before paraffin embedding. Serial tissue sections (2 μm thick) were deparaffinized in xylene, rehydrated with a series of ethanol solutions (100% to 50%), and incubated in a 0.3% hydrogen peroxide solution for 20 min to block endogenous peroxidase activity. Sections were then rinsed with tap water, washed with phosphate-buffered saline (PBS) and subjected to antigen retrieval by boiling in a Tris/EDTA (pH 9.0) solution for 20 min. Subsequently, the sections were cooled, rinsed with tap water and PBS, and incubated with normal serum at room temperature for 30 min, followed by hematoxylin and eosin (H&E) staining or IHC. For IHC, the sections were incubated with MMP-9 or Madcam1 primary antibodies overnight at 4 °C. After rinsing with PBS three times, the slides were incubated for 30 min with HRP-conjugated secondary antibodies. Signal was developed using a 3′-diaminobenzidine (DAB) kit (Solarbio, Beijing, China).

### Statistical analysis

Data were analyzed using unpaired Student’s t test and are expressed as mean ± SD. P < 0.05 was considered statistically significant. All graphs and statistical calculations were performed using GraphPad Prism software (Version 6.0; La Jolla, CA, USA).

## Supplementary Material

Supplementary Figures

Supplementary Table 1
