# Supplementary material for: TRIM59 loss in M2 macrophages promotes melanoma migration and invasion by upregulating MMP-9 and Madcam1
Source: Aging (Albany NY). 2019 Oct 10;11(19):8623–41. doi: 10.18632/aging.102351 (PMC6814609; doi:10.18632/aging.102351)
Supplement: Supplementary Figures [file aging-11-102351-s002.pdf]

SUPPLEMENTARY FIGURES

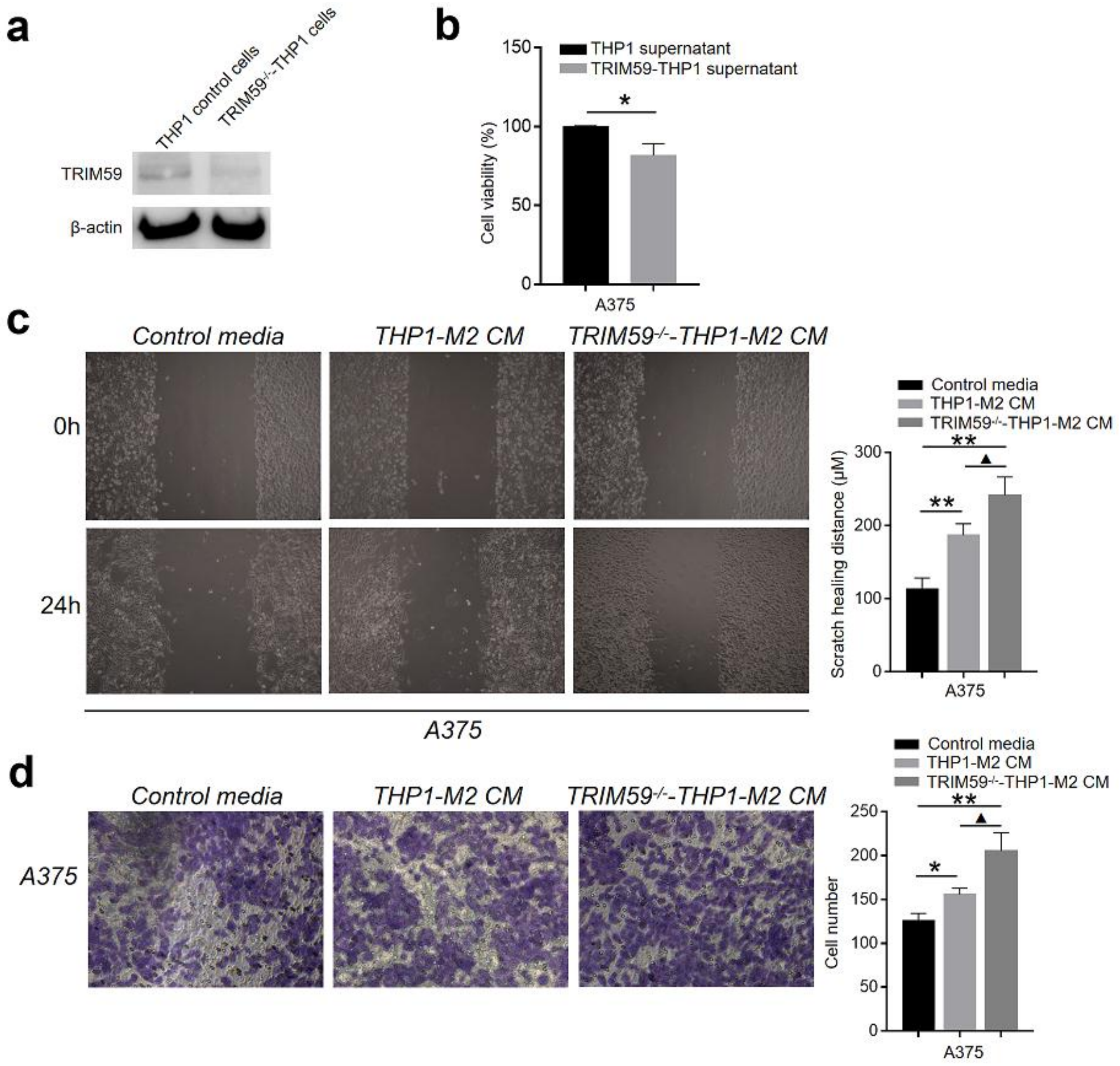

Supplementary Figure 1. (**A**) Expression of TRIM59 in THP1 cells transfected with *TRIM59*-shRNA, as assessed by western blotting. (**B**) CCK-8 showing that TRIM59 overexpressed THP1 has a cytotoxicity of 18.25% on A375 human melanoma cells. (**C**) Wound healing assay results showing the migratory ability of A375 cells in response to CM from Control meidia, THP1-M2 macrophage cultures or TRIM59$^{-/-}$-THP1-M2 macrophage cultures. Data are represented as mean ± SD. **p<0.01, compared with control media; ▲p<0.05, TRIM59$^{-/-}$-THP1-M2 CM vs. THP1-M2 CM. (**D**) Transwell assays results showing the invasive ability of A375 cells In response to CM from Control meidia, THP1-M2 macrophage cultures or TRIM59$^{-/-}$-THP1-M2 macrophage cultures. Data are represented as mean ± SD. *p<0.05, **p<0.01, compared with control media; ▲p<0.05, TRIM59$^{-/-}$-THP1- M2 CM vs. THP1-M2 CM.

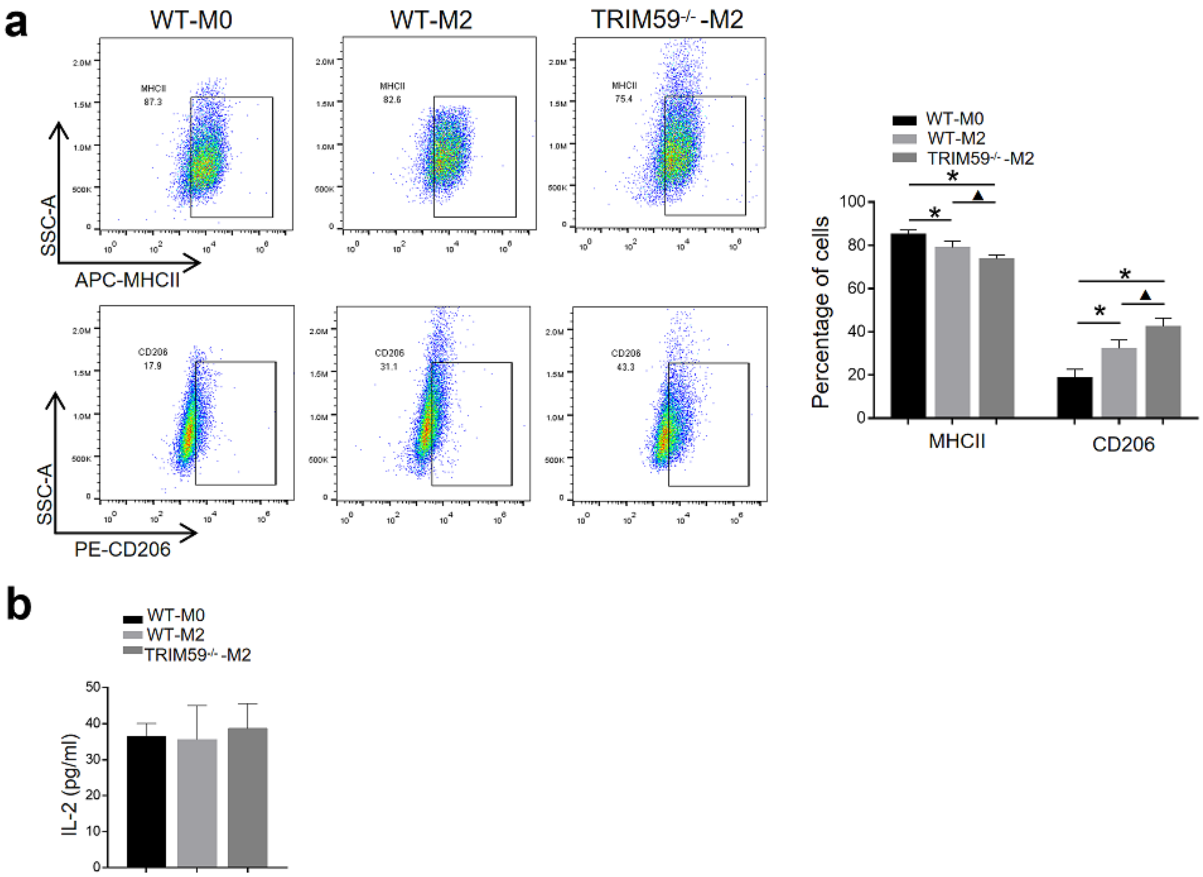

Supplementary Figure 2. (**A**) Flow cytometry analysis of macrophage subpopulations based on MHCII and CD206 expression. Data are represented as mean ± SD. **p*<0.05, compared to WT-MO macrophages; ▲p<0.05, TRIM59$^{-/-}$-M2 vs. WT-M2. (**B**) ELISA detection of IL2 in culture supernatants from WT-MO, WT-M2, and TRIM59$^{-/-}$-M2 macrophages. Data are represented as mean ± SD.

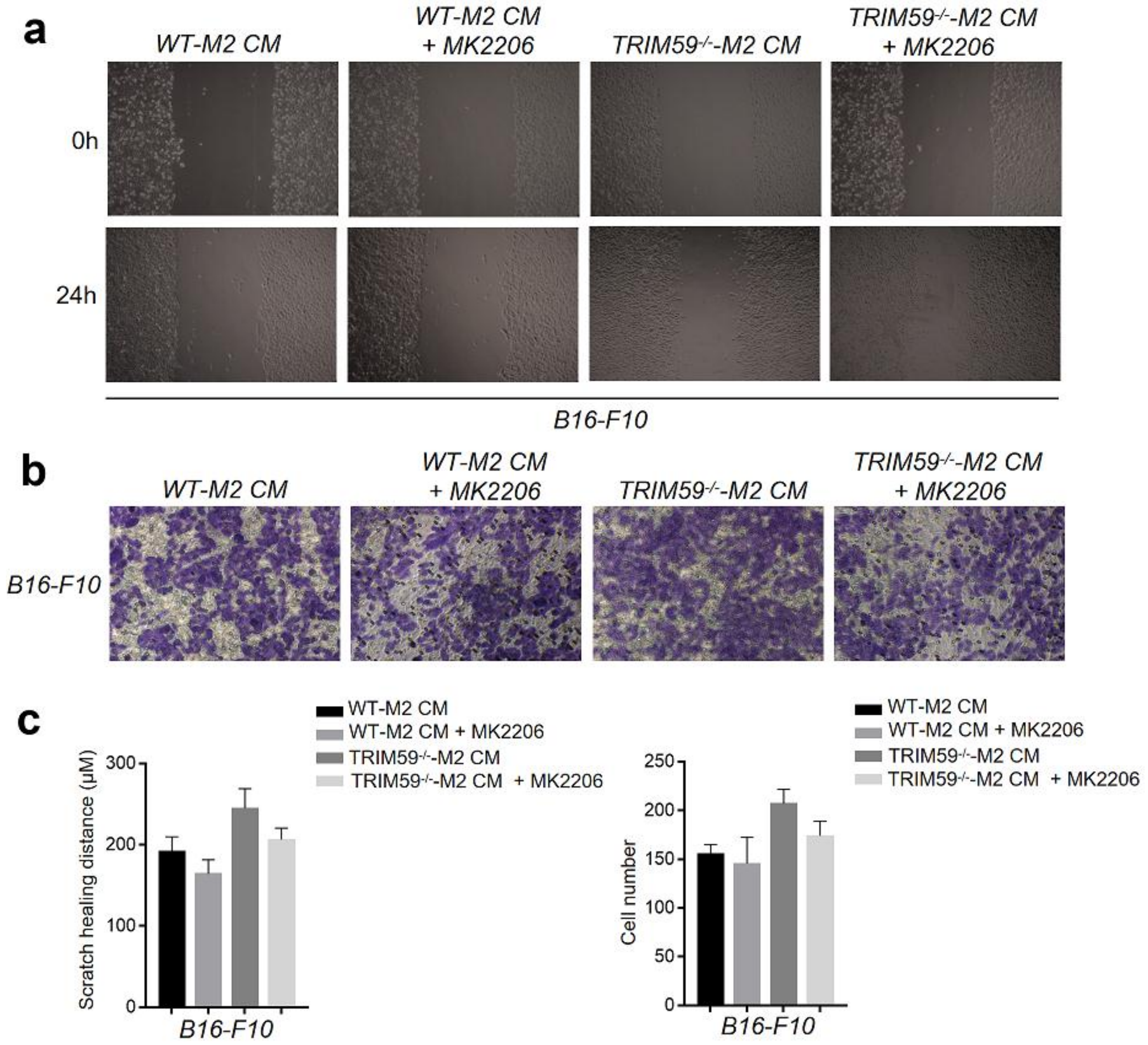

Supplementary Figure 3. (**A**) Wound healing assay results showing the migratory ability of B16-F10 cells in response to CM from M2 macrophage cultures in the presence of the Akt inhibitor MK2206. (**B**) Transwell assays results showing the invasive ability of B16-F10 cells in response to CM from M2 macrophage cultures in the presence of the Akt inhibitor MK2206. (**C**) Statistical analysis of (**A**) and (**B**). Data are represented as mean ± SD.
