# Supplementary material for: TRIM59 loss in M2 macrophages promotes melanoma migration and invasion by upregulating MMP-9 and Madcam1
Source: Aging (Albany NY). 2019 Oct 10;11(19):8623–41. doi: 10.18632/aging.102351 (PMC6814609; doi:10.18632/aging.102351)
Supplement: Supplementary Table 1 [file aging-11-102351-s001.pdf]

SUPPLEMENTARY TABLE

Supplementary Table 1. Primer sequences used for qPCR analyses.

	Forward primer (5′-3′)	Reverse primer (5′-3′)
Ccl2	GCTACAAGAGGATCACCAGCAG	GTCTGGACCCATTCCTTCTTGG
Cxcl3	TGAGACCATCCAGAGCTTGACG	CCTTGGGGGTTGAGGCAAACTT
Erbb4	CAAAGCCAACGTGGAGTTCATGG	CTGCGTAACCAACTGGATAGTGG
Mmp12	CACACTTCCCAGGAATCAAGCC	TTTGGTGACACGACGGAACAGG
Cxcl5	CCGCTGGCATTTCTGTTGCTGT	CAGGGATCACCTCCAAATTAGCG
Cxcl2	CATCCAGAGCTTGAGTGTGACG	GGCTTCAGGGTCAAGGCAAACT
Ccl20	GTGGGTTTCACAAGACAGATGGC	CCAGTTCTGCTTTGGATCAGCG
Mmp9	GCTGACTACGATAAGGACGGCA	TAGTGGTGCAGGCAGAGTAGGA
Cxcl1	TCCAGAGCTTGAAGGTGTTGCC	AACCAAGGGAGCTTCAGGGTCA
Madcam1	TGTCAGACACAGGCACTCCTGT	CTGTCCAGGTACAAGGAACTCC
